# Polyethylene Terephthalate Glycolysis: Kinetic Modeling and Validation

**DOI:** 10.3390/polym17162246

**Published:** 2025-08-20

**Authors:** Maja Gabrič, Žan Lavrič, Martin Schwiderski, Laureline Marc, Erik Temmel, Miha Grilc, Blaž Likozar

**Affiliations:** 1 Department for Catalysis and Chemical Reaction Engineering, National Institute of Chemistry, Hajdrihova 19, SI-1000 Ljubljana, Slovenia; maja.gabric@ki.si (M.G.); miha.grilc@ki.si (M.G.); blaz.likozar@ki.si (B.L.); 2 Graduate School, University of Nova Gorica, Vipavska 13, SI-5000 Nova Gorica, Slovenia; 3 Sulzer Chemtech Ltd., Neuwiesenstrasse 15, 8401 Winterthur, Switzerland; martin.schwiderski@sulzer.com (M.S.); laureline.marc@sulzer.com (L.M.); erik.temmel@sulzer.com (E.T.); 4 Faculty of Polymer Technology, Ozare 19, SI-2380 Slovenj Gradec, Slovenia

**Keywords:** PET depolymerization, glycolysis, homogeneous catalysis, kinetic modeling

## Abstract

In this study, a comprehensive investigation of PET glycolysis has been performed. The research included the development of analytical techniques, experiments with different reactor systems, and the development of mathematical models to understand the kinetics of the process. Quantitative HPLC analysis was adopted and optimized for the detection of BHET, while the size-exclusion chromatography method was developed to determine the molecular weight distribution of solid PET residues. Over 33 experiments were performed with magnetically coupled shaft-stirred Amar reactors, resulting in more than 300 experimentally determined BHET concentration points at various reaction times, temperatures, catalyst concentrations, and ethylene glycol-to-PET ratios. Afterwards, a kinetic model was developed to describe the observed phenomena with a validation step.

## 1. Introduction

Polyethylene terephthalate (PET) is a versatile thermoplastic polymer that is widely used in the packaging industry, in the manufacturing of stretch bottles, and in textile fibers. With the increasing importance of government regulations and measures to promote the circular economy, PET recycling has become an important area of research in the past decades [1]. Currently, mechanical recycling of PET is already performed on an industrial level; however, each recycling leads to a degradation of mechanical properties, meaning that the use of mechanical recycling alone cannot eliminate the need for virgin PET material from fossil resources [2]. From a circular economy perspective, chemical recycling thus offers a closed loop that could significantly reduce the demand for virgin TPA through the continuous depolymerization of PET material, rendering higher value of waste PET material. Chemical recycling of PET offers promising solutions by producing precursors such as dimethyl terephthalate (DMT) by methanolysis [3,4] or bis (2-hydroxyethyl) terephthalate (BHET) by glycolysis [5,6], which can be used to synthesize purified terephthalic acid (TPA) through hydrolysis [7,8]. Alternatively, PET can be depolymerized to TPA and EG by base hydrolysis [9], by ZSM-5 zeolites [10], or also with the use of deep eutectic solvents [11].

A major advantage of glycolysis is its compatibility with conventional PET production plants so that recovered BHET can be mixed with new BHET (produced from terephthalic acid and ethylene glycol). In addition, the monomers and oligomers recovered from glycolysis can be reused to synthesize other high-value polymers such as unsaturated polyesters, polyurethane foams, and polyisocyanurate foams [12]. Glycolysis normally takes place at temperatures between 180 and 240 °C and can be accelerated with metal catalysts [13]. Without a catalyst, the selectivity for BHET also decreases, which makes it difficult to purify the reaction mixture (dimers and trimers) using conventional separation methods.

While there are numerous experimental studies regarding PET glycolysis with different catalysts [7,14,15,16,17], the kinetic aspect has not been fully elucidated. The main problem is that depolymerization involves scission of long linear PET chains to shorter counterparts. In other words, a mass balance equation for each oligomer/polymer chain, which can undergo scission at every ester bond it has, is rather impractical. Therefore, various simplifications are required when constructing a model to describe the rate of PET glycolysis and extract the relevant data in terms of reaction temperature and time, initial particle size, and stirring rate. For example, Chen et al. [15] performed the reactions in a temperature range from 230 to 280 °C at different zinc acetate dihydrate catalyst concentrations. The kinetic model was based on ethylene glycol (EG) consumption, during the reaction. They discovered that the catalyst influences the activation energy barrier (*Ea*) of the reaction. The latter was determined to be 85 and 108 kJ mol^−1^ for catalyzed and uncatalyzed depolymerization, respectively. They also discovered that the effect of the amount of the catalyst on the reaction was linearly proportional to the reaction rate, but after a certain threshold value, the reaction rate was not further increased. Later, Viana et al. [16] included different sites at which polymer can be cleaved. They postulated that ester bonds at the end of the chain, the ones across the benzene ring from the end of the chain, and the ones in between can describe the depolymerization of BHET. The analysis showed two regions (lower and higher temperature regions), each with a different activation barrier. In the lower temperature region (179–180 °C), *Ea* was determined to be 99 kJ mol^−1^, which corresponded with the values reported in the literature. In the higher temperature region (180–200 °C), the activation barrier decreased to 41.7 kJ mol^−1^. López-Fonseca [18] later discovered that the depolymerization of PET flakes to BHET occurs with consecutive reactions, where PET solids are irreversibly depolymerized into oligomers and dimers, which are then in an equilibrium with BHET in the liquid phase. The catalyst used was sodium carbonate in a temperature range from 165 to 196 °C at a fixed EG-to-PET *w*/*w* ratio of 2.5. The highest BHET yield determined was around 80%, and longer reaction times did not improve this value, confirming the reversibility from dissolved oligomers and dimers to BHET. However, they did not study depolymerization at higher EG-to-PET *w*/*w* ratios, which would validate their reversible kinetic model, since at higher EG-to-PET *w*/*w* ratios, ethylene glycol concentration is higher, forcing equilibrium towards BHET. In their study they also determined the activation energy for depolymerization to be around 185 kJ mol^−1^. Wei Li and coworkers studied depolymerization of PET fibers [14]. Similarly, as previous researchers, they confirmed that above 1.0 wt.% of Zn/Al catalyst, the reaction rate is not improved. Their kinetic analysis showed a lower activation energy barrier of 79.3 kJ mol^−1^, which is lower than for PET flakes, accordingly because the PET material in fibers is composed of shorter polymeric chains and also has a higher surface area.

The main motivation for our study was to investigate the glycolysis of polyethylene terephthalate using a zinc acetate dihydrate catalyst, systematically exploring important process parameters that were only separately considered in previous studies. These parameters included temperature, EG-to-PET *w*/*w* ratio, particle size, stirring speed, catalyst concentration, dynamic EG addition, and in situ water removal. In contrast to previous work, where process conditions were often not extensively varied, our research aimed to bridge this gap by varying process conditions and advanced product analysis techniques. A robust kinetic model for PET glycolysis was developed and experimentally validated. A wide range of conditions were investigated, including temperatures from 150 to 190 °C, zinc acetate dihydrate concentrations from 0.6 to 6 wt.% (based on the added PET mass), and mass ratios of EG-to-PET from 2.5 to 5. Reaction times were systematically varied, with additional experiments of shorter duration (60 min, including heating) to capture partially depolymerized PET flakes. These residual flakes were analyzed by size exclusion chromatography (SEC), which provides information on the changes in molecular weight during the depolymerization process. The comprehensive approach of our study ensures that the kinetic model is both robust and reliable, providing valuable insights to optimize PET glycolysis under different process conditions.

## 2. Material and Methods

Materials, equipment, and experimental procedures, as well as the methodology for kinetic modeling used, are described in this section and in the Supplementary Material.

### 2.1. Reactor Equipment

Experiments were performed in a high-pressure autoclave system (Amar Equipment Pvt. Ltd., Mumbai, India). Each batch system consists of a 250 mL vessel (inner diameter 67 mm and height 80 mm), with a magnetically driven Rushton turbine impeller (diameter of 30 mm, placed 14 mm above the autoclave bottom). The autoclave system is equipped with a separate internal and external cooling system that ensures proper temperature regulation inside of each autoclave and for outer parts (sampling lines, head of the impeller). Initial PET material was provided by Sulzer Chemtech Ltd. (Winterthur, Switzerland). Reactions were carried out at three different ethylene glycol (EG, Sigma Aldrich, Taufkirchen, Germany, 99%) EG-to-PET mass ratios, namely 5.0 and 2.5. Described stepwise, firstly the reactor was charged with the desired weight of PET flakes, EG, and catalyst (uncatalyzed reactions did not include catalyst). Then, the catalyst was sealed and mounted to the system with a heater. The reactor underwent three cycles of nitrogen (N_2_, Messer, Bad Soden, Germany) flushing before being heated up, while maintaining a stirring speed of 1000 rpm. The sampling was performed by opening one of the reactor’s outlets and discarding the liquid mixture with a syringe. This kind of sampling was necessary because sampling through the dip-tube was not possible due to the high viscosity of the mixture and PET flakes, which constantly clogged the tube. At the end, the reactor was cooled back to room temperature. In the experiments for the molecular weight distributions, described in Section 2.2, the procedure to prepare samples is described in the Supplementary Information. A series of reactions were then carried out at three different temperatures (Figure S2, 150, 170, and 190 °C), three different catalyst concentrations of zinc acetate dihydrate (Merck, Darmstadt, Germany) (6.0, 3.0, and 0.6 wt.% per PET material in the experiment), and experiments without added catalyst. This systematic variation in both temperature and catalyst concentration enabled a thorough investigation of the reaction’s dependence on these critical parameters. All the reaction parameters are described in Table S2. For quantification of BHET and MHET (monohydroxyethyl terephthalate) after the glycolysis, high-performance liquid chromatography (HPLC) was used and is presented in the Supplementary Information. Experiments tailored for SEC analysis, different particle sizes of initial material, stirring variation, and experiments with water addition are described in Supplementary Information. (BHET from Sigma Aldrich with purity 96% and MHET from BLD pharm with 95% purity was used for calibration (Figure S1)).

### 2.2. Size Exclusion Chromatography

Size exclusion chromatography (SEC) was performed, to determine molecular weight distribution of solid PET residue. SEC is an analytical method employed to separate organic polymer mixtures by segregating analytes according to their size, wherein molecules are partitioned based on their exclusion from pores within the column packing material. Larger analytes are eluted initially, whereas smaller molecules exhibit greater interaction with the stationary phase, leading to their later elution [19].

Analysis was conducted with a ThermoFisher Scientific Ultimate 3000 instrument (Waltham, MA, USA) using an Agilent 2 × PL HFIP Column (250 mm × 4.6 m) and with a PL HFIP gel Guard Column (Agilent, Santa Clara, CA, USA). The best separation of compounds of interest was achieved with the method, which involved a 0.3 mL min^−1^ flow rate of 1,1,1,3,3,3-Hexafluoro-2-propanol (HFIP) (Sigma Aldrich, Taufkirchen, Germany) with 20 mM Sodium Trifluoroacetate (NaTFA) (Sigma Aldrich, Germany). The column was kept at 30 °C, and the injection volume was 10 μL. An RI detector was used for quantification of compounds. For the calibration first, PET standards were used (PSS Polymer Standard Service, Agilent, Mainz, Germany) (3500 Da–120,000 Da of Mw). However, due to the low solubility of the PET standard materials with higher Mw in the prepared mobile phase, the calibration curve was constructed using polymethyl methacrylate (PMMA) (PSS Polymer Standard Service, Agilent, Mainz, Germany) in the range of 651 Da to 517,000 Da as a surrogate compound for PET. Several methodologies have also employed this polymer for calibration purposes instead of PET standards [20]. SEC analysis was performed on initial PET flake material and residual PET solids, obtained after experiments were performed at 150, 170, and 190 °C; 0.6, 3.0, and 6.0 wt.% of catalyst; EG-to-PET *w*/*w* ratio of 5 and 2.5; and reaction time of 60 min. The molecular weights were calculated with the software PSS WIN GPC (UniChrom, V 8.32, Mainz, Germany). After the reaction, residual PET material from the reactor was weighed and the liquid phase was analyzed for BHET quantification. (The results, shown in Table S1 indicate that the margin of error in the determination of PET molecular weights with the PMMA standards is approximately 25% underestimated).

### 2.3. Kinetic Modeling

To develop a kinetic model to describe the experiments, a few simplifications have been made. First, since the concentration of PET cannot be determined in a classic way, the reactant concentration is calculated by dividing the mass of PET by the molecular weight of the monomer unit, calculated by the following Equation (1):

(1) $$C_{solid\;PET}=\frac{m_{PET}}{{Mw}_{monomer}}$$

where m_PET_ is the mass of PET in the experiment and Mw_monomer_ is the molecular mass of the monomer, which is 210.182 g mol^−1^. This gave us an initial *n* of monomers, “locked” inside PET chains. Each monomer can give exactly one BHET molecule, with the addition of one ethylene glycol during the reaction. Solid PET is firstly converted to oligomers/dimers, which are denoted as intermediates. Hydrolysis to MHET was not accounted for in the current model, since the amount of MHET is fairly low. Moreover, if all PET is converted to only BHET, the theoretical yield of BHET [16] can be calculated by Equation (2), written as follows:

(2) $$m_{theoretical\;yield\;of\;BHET}=m_{PET}\times 1.323$$

This means that if 20 g of pure PET is charged into the reactor, the mass of BHET at the end of the reaction should not exceed 26.46 g [16].

From this point on, the reaction rate equations are standard kinetic equations. Reaction rate constants were set to follow the Arrhenius law, calculated by Equation (3), written as follows:

(3) $$k_{i}(T_{2})=k_{i}(T_{1})\times exp\left(\frac{{Ea}_{i}}{R}\times \left(\frac{1}{T_{1}}-\frac{1}{T_{2}}\right)\right)$$

Reaction rate equations were adjusted for uncatalyzed and catalysed reactions. Equations (4)–(8) represent the reaction rate of each step, written as follows:

Equation (4)

(4) $$r_{1}=k_{1}\times C_{solid\;PET}$$

Equation (5)

(5) $$r_{2}=k_{2}\times C_{solid\;PET}\times {C_{catalyst}}^{\alpha }$$

Equation (6)

(6) $$r_{3}=k_{3}\times C_{Intermediates}$$

Equation (7)

(7) $$r_{4}=k_{4}\times C_{Intermediates}\times {C_{catalyst}}^{\beta }$$

Equation (8)

(8) $$r_{5}=k_{5}\times C_{BHET}\times exp\left(S_{1}\times \frac{1}{ratio}\right)$$

Equation (8) represents the backward reaction. They are multiplied with an exponential function, as in the previous model, which affects the reaction rate in the following manner: If the EG-to-PET *w*/*w* ratio is lower, the backward reactions are faster, and thus, the final yield of BHET is lower than anticipated. If the ratio is higher, the yield of BHET will be closer to the theoretical yield.

The mass balance equations for the reversible model are shown in the following Equations (9)–(11):

Equation (9)

(9) $$\frac{dC_{solid\;PET}}{dt}=-r_{1}-r_{2}$$

Equation (10)

(10) $$\frac{dC_{Intermediates}}{dt}=+r_{1}+r_{2}-r_{3}-r_{4}+r_{5}$$

Equation (11)

(11) $$\frac{dC_{BHET}}{dt}=+r_{3}+r_{4}-\;r_{5}$$

The mathematical analysis was carried out using MATLAB 2021b (The Mathworks, Inc., Natick, MA, USA) software. The concentration profiles were solved as a function of time using the ODE15s solver. The regression analysis was performed with the Nelder–Mead function fminsearch. The regression was constrained to ensure that the activation energies of competing uncatalyzed reactions were not lower than those of their catalyzed counterparts.

## 3. Results and Discussion

### 3.1. Size Exclusion Chromatography

**Figure 1:**
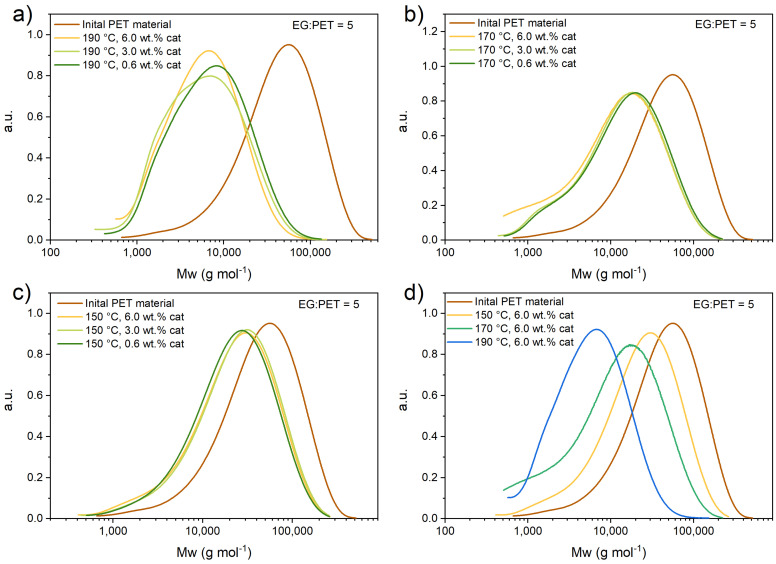
SEC experiments at (**a**) 190, (**b**) 170, and (**c**) 150 °C, with 6.0, 3.0, and 0.6 wt.% of catalyst added. EG-to-PET *w*/*w* ratio of 5 and reaction time of 60 min; (**d**) SEC experiments at 190, 170, and 150 °C with catalyst 6.0 wt.%, EG-to-PET *w*/*w* ratio of 5, and reaction time of 60 min. Reaction conditions are presented in Table S2, Entries 19–27.

**Figure 2:**
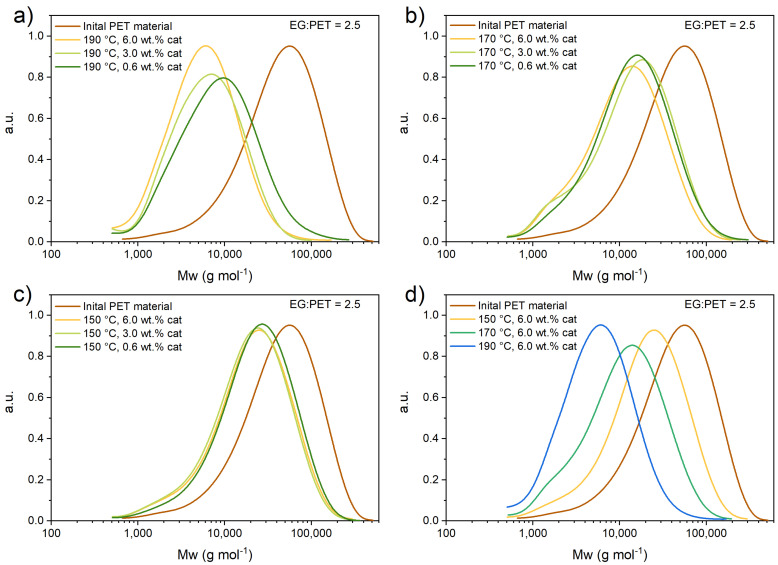
SEC experiments at (**a**) 190, (**b**) 170, and (**c**) 150 °C with 6.0, 3.0, and 0.6 wt.% of catalyst added EG-to-PET *w*/*w* ratio of 2.5 and reaction time of 60 min. (**d**) SEC experiments at 190, 170, and 150 °C with catalyst 6.0 wt.%, EG-to-PET *w*/*w* ratio of 5, and reaction time of 60 min. Reaction conditions are presented in Table S2, Entries 28–36.

To determine the molecular weight distribution of initial PET flakes and residual PET flakes (after the reaction, as described in ESI), SEC analysis was performed. Figure 1 and Figure 2 represent the molecular weight distribution of the initial PET sample and each residual fraction at set experimental conditions. Table S3 summarizes the results obtained by SEC-HPLC for EG-to-PET *w*/*w* ratios at different conditions for each experiment. The table shows the calculated average molecular weight of the filtered solid PET residue after the reaction, the mass of the filtered solid PET residue after the reaction, and the measured liquid product (from the HPLC results). (Figures S3–S5 show the concentration vs. time profiles, which were also use within regression analysis for kinetic parameter estimation. These experiments are actually the short reaction time experiments and the residual solid PET was used for SEC analysis.)

As can be seen in Figure 1, both temperature and catalyst presence affect the *M*_w_ distribution. At 190 °C and 6.0 wt.% of catalyst, there were only 2.7 g of residual solid PET (20 g, charged to the reactor initially). In the latter, the peak shifts to lower *M*_w_ and narrows down, suggesting that during depolymerization a scission reaction to di-, tri-, and oligomers occurs.

**Figure 3:**
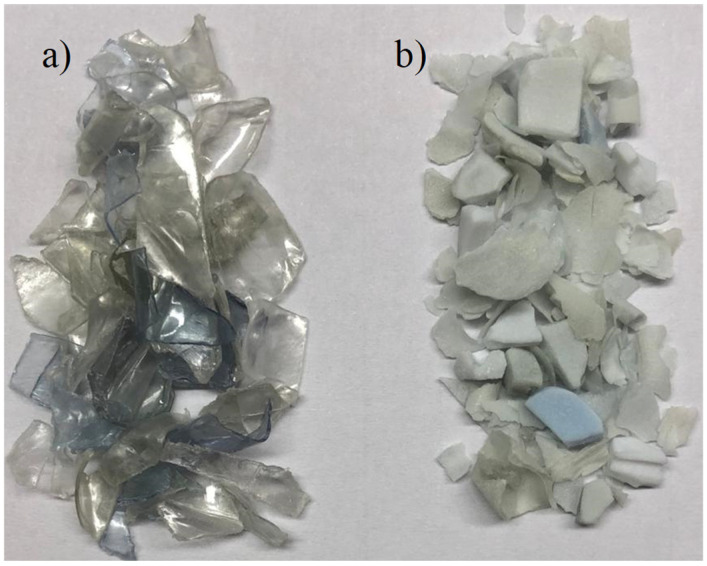
Visual representation of (**a**) starting PET flakes and (**b**) PET residues.

To support the statement, the initial flakes and flake residues after the reaction have different physical properties. Their opacity is higher, and they become more brittle [21]. This is shown in Figure 3.

The same was performed for EG-to-PET with a *w*/*w* ratio of 2.5. Figure 2 shows PET *M*_w_ distribution for experiments at 190, 170, and 150 °C, various catalyst wt.%, an EG-to-PET *w*/*w* ratio of 2.5, and a reaction time of 60 min.

Experiments performed at an EG-to-PET *w*/*w* ratio of 2.5 show a similar PET flake depolymerization trend as experiments performed at an EG-to-PET *w*/*w* ratio of 5. The main difference is that the reaction is slower due to the lower concentration of EG. As can be seen in Table S3, the residual mass of PET flakes is higher (~2-fold) at a lower PET ratio of 2.5. This further suggests a faster flake depolymerization, if a higher EG-to-PET *w*/*w* ratio is used. From the figures above, one can observe that temperature has a much greater effect on the Mw distribution of solid PET flakes than the catalyst concentration. Catalyst concentration has no actual effect on the distribution at the same temperature. This is consistent with study [14,18], where temperature also has a much more pronounced effect on the reaction.

### 3.2. Particle Size and Stirring Speed Effect

**Figure 4:**
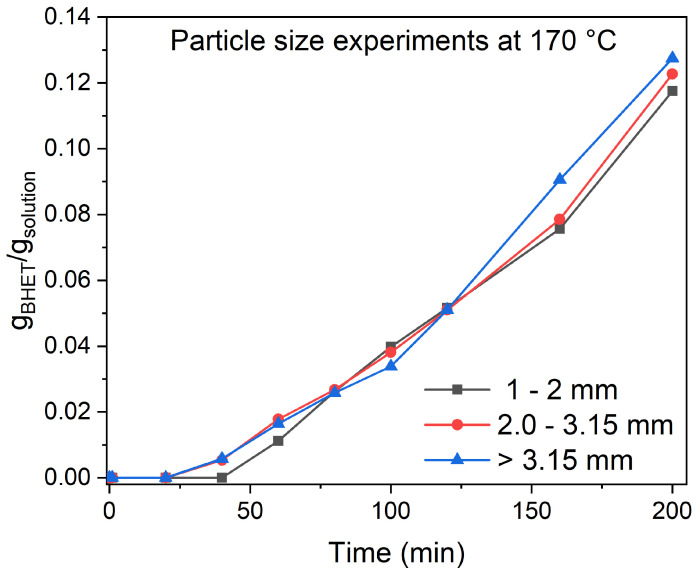
Experimental runs for three different particle sizes and the following experimental conditions: 170 °C, EG-to-PET *w*/*w* ratio of 5, 0.6 wt.% of catalyst, and 1000 rpm. Reaction conditions are presented in Table S2, Entries 37–39.

To determine the effect of PET flake size, experiments were performed at 3 different PET sizes. The initial PET material has been cut in a cutting mill to three different flake sizes (1–2 mm, 2–3.15 mm, and >3.15 mm). The experiments were performed at 170 °C, with an EG-to-PET *w*/*w* ratio of 5, 0.6 wt.% of catalyst, and 1000 rpm. The results are shown in Figure 4.

The different particle sizes do not affect the final BHET yield, which is 0.123 g BHET/g solution ± 0.004 for all three sizes. The particle size effect on the final BHET yield was studied by López-Fonseca et al. [18] and showed that the particle size affects the final BHET yield when the particle size is above 0.71 mm, while conversion rates for sizes between 0.14 and 0.35 mm were similar. The discrepancy between their findings and ours can be explained by key differences in experimental conditions, including the catalyst type (sodium carbonate in their study versus zinc acetate dihydrate in ours), EG-to-PET *w*/*w* ratio (2.5 in their study versus 5 in ours), reaction temperature (175 °C in their study versus 150 °C in ours), stirring speed (600 rpm in their study versus 1000 rpm in ours), and particle size range examined.

Nonetheless, our data suggest that for an efficient kinetic description of the process, the PET flake particle size does not affect the overall conversion rate to such an extent, suggesting that particle size does not have to be included in the kinetic model as a descriptor.

**Figure 5:**
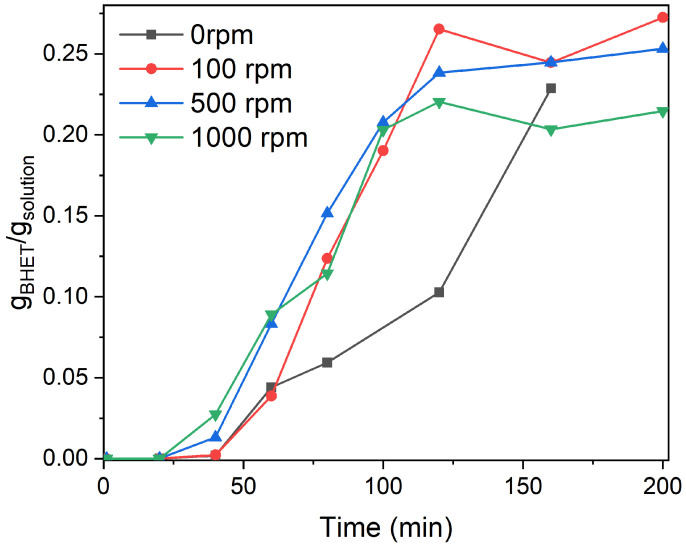
Stirring speed variation at 190 °C, EG-to-PET *w*/*w* ratio of 5, 0.6 wt.% of catalyst. Reaction conditions are presented in Table S2, Entries 40–43.

Another important descriptor for kinetic modeling is the stirring speed of the reactor, affecting the external mass transfer, which is, in the case of PET glycolysis, the transfer of ethylene glycol to the PET flake surface. To test how stirring speed affects BHET yield during depolymerization, experiments at 0, 100, 500, and 1000 rpm were performed (Figure 5).

Surprisingly, stirring speed had less influence on the conversion rate of PET than expected. The concentration profiles of BHET at 100, 500, and 1000 rpm are practically the same, reaching equilibrium after 120 min of reaction. This is partially consistent with a previous study [18], where they also observed a marginal increase in activity when stirring speed was in the range from 200 to 800 rpm. Their results suggest that at lower stirring rates (50 rpm), the final yield was considerably lower, although BHET yield after 3 h of reaction (EG-to-PET *w*/*w* ratio of 2.5, 185 °C, 100:1 molar ratio of PET to catalyst) at 50 rpm was only 10% lower than at 800 rpm.

As mentioned above, in our case an EG-to-PET *w*/*w* ratio of 5 was applied. To determine external mass transfer resistance, we performed a reaction at 190 °C, EG-to-PET *w*/*w* ratio of 5, and 0.6 wt.% of the catalyst, without stirring. Notably, the BHET yield after 150 min of reaction was equivalent to that observed in experiments with agitation. This observation was further corroborated by visual inspection of the reactor post-reaction, which revealed an absence of PET flakes within the reactor. This interesting revelation showed us that there is very low external mass transfer resistance for EG, which is usually not encountered in solid–liquid reaction systems. However, EG is not only a reactant but also a solvent, which means that there is no other inert medium through which it has to diffuse to reach the solid scission sites on PET flakes. Therefore, omitting diffusion in the liquid phase is a reasonable simplification for later kinetic modeling. Consequently, as EG functions as both solvent and reactant, diffusion in the liquid phase need not be incorporated into kinetic modeling.

### 3.3. Glycolysis Kinetics

As it was suggested from SEC analysis, the “dissolution” step of PET is in fact a direct depolymerization from the solid PET flakes. The molecular weight of polymeric chains inside residual flakes become shorter during the reaction. With this in mind, PET is not dissolving but is directly depolymerized to oligomers/dimers, which are accounted for as *intermediates* in our model. The latter is then finally glycolyzed to BHET. Experiments with different particle sizes and stirring speeds also allowed us to make two simplifications when constructing a kinetic model, namely omitting the particle size effect in terms of flake surface area and the diffusion of EG to the PET flake surface, since it is also a solvent and is uniformly distributed throughout the reaction mixture.

#### 3.3.1. Catalyst Effect on the Reaction

**Figure 6:**
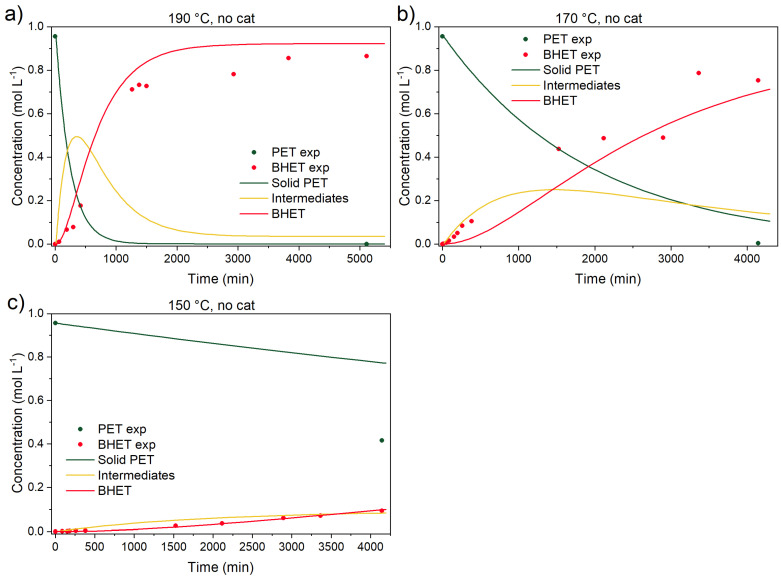
Uncatalyzed PET glycolysis at (**a**) 190, (**b**) 170, and (**c**) 150 °C. Reaction conditions: EG-to-PET *w*/*w* ratio of 5, 1000 rpm. Reaction conditions are presented in Table S2, Entries 4, 8, and 12.

It is well known that PET glycolysis does not require a catalyst for a successful depolymerization [15], but the reaction times to achieve the final BHET yield are, consequently, longer. This can be seen in Figure 6, where the time to reach the final BHET yield of 95% is above 3000 min. At 170 °C, the reaction is a bit slower, yielding 80% of BHET, while at 150 °C, BHET yield is 14% after 4000 min of reaction.

**Figure 7:**
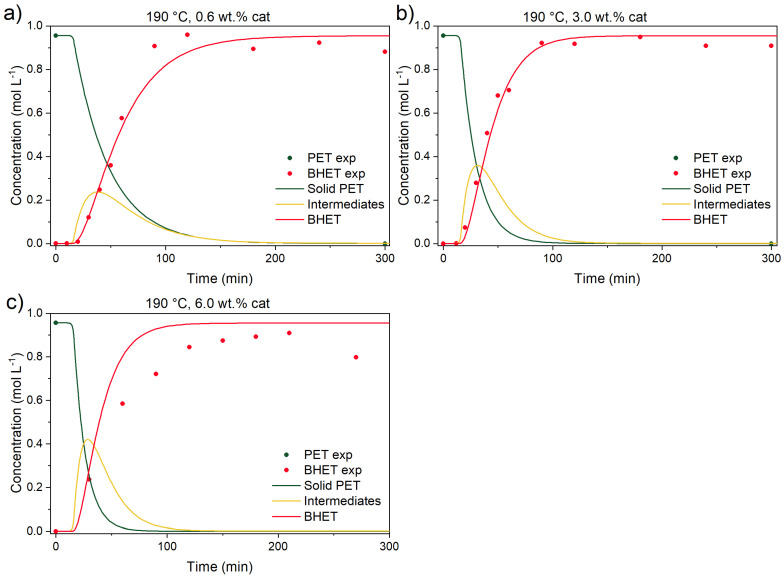
Catalyzed PET glycolysis at 190 °C with (**a**) 0.6, (**b**) 3.0, and (**c**) 6.0 wt.% catalyst. Reaction conditions: EG-to-PET *w*/*w* ratio of 5, 1000 rpm. Reaction conditions are presented in Table S2, Entries 1–3.

**Figure 8:**
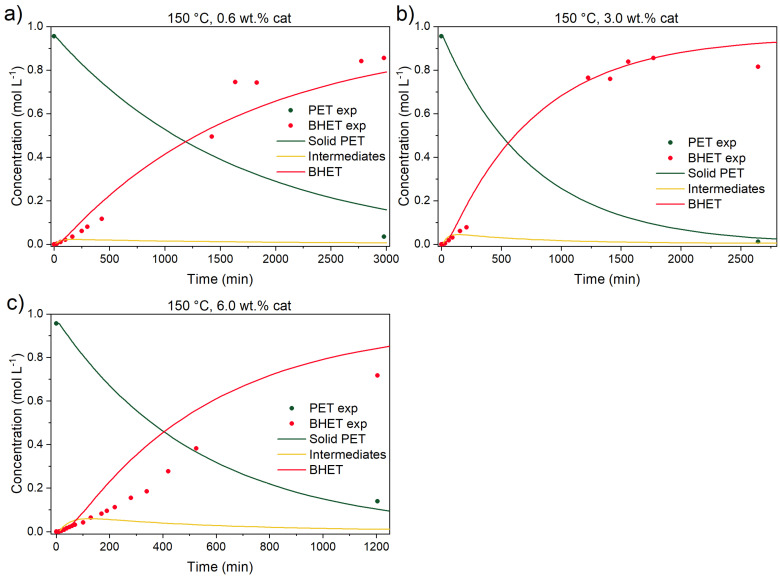
Catalyzed PET glycolysis at 150 °C with (**a**) 0.6, (**b**) 3.0, and (**c**) 6.0 wt.% catalyst. Reaction conditions: EG-to-PET *w*/*w* ratio of 5, 1000 rpm. Reaction conditions are presented in Table S2, Entries 9–11.

Previous kinetic studies [15,18] already partially addressed the uncatalyzed depolymerization contribution. The first objective was to determine each reaction’s contribution to the overall PET conversion. In Figure 7 and Figure 8, catalyzed reactions at three different temperatures (150, 170, and 190 °C) and catalyst concentrations (0.6, 3.0, and 6.0 wt.% of the initial reaction mixture’s mass) are shown.

**Figure 9:**
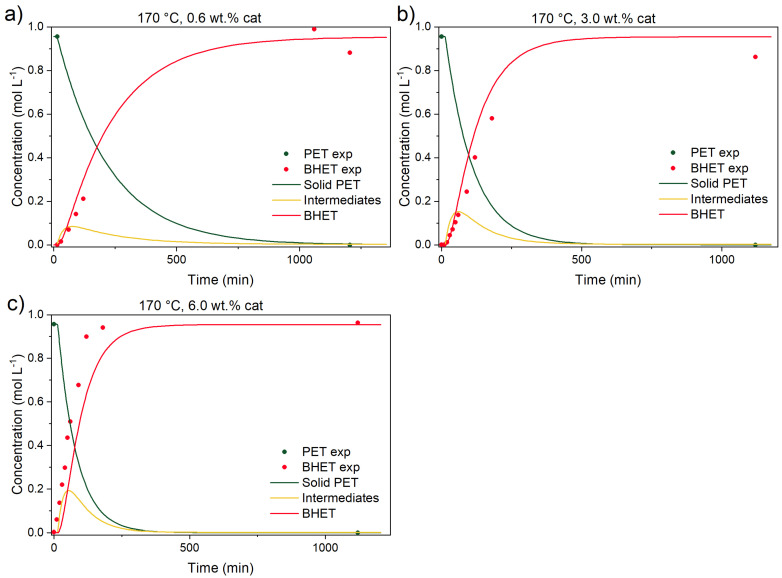
Catalyzed PET glycolysis at 170 °C with (**a**) 0.6, (**b**) 3.0, and (**c**) 6.0 wt.% catalyst. Reaction conditions: EG-to-PET *w*/*w* ratio of 5, 1000 rpm. Reaction conditions are presented in Table S2, Entries 5–7.

It can be seen that the addition of the catalyst improves the rate of conversion immensely at 190 °C. In other words, final BHET yield is achieved in 100 min instead of 5000 min (50-fold improvement). Comparing BHET yield between the uncatalyzed and catalyzed reactions at 190 °C after 100 min, the former produces 3.35% of BHET while the catalyzed reaction produces 99% of BHET. The same comparison can be drawn from reference [14], where BHET yields, after 20 min of reaction, for uncatalyzed and catalyzed reactions are ~4 and 80%, respectively. Similar observations can be seen in Figure 8 and Figure 9 at 150 and 170 °C.

From the figures above, it can be seen that although the addition of the catalyst improves all reaction rates (dissolution of BHET and conversion of intermediates to BHET), the added amount of the catalyst hardly improves the rate of PET conversion. Similar observations were detected also in other studies, but direct comparison is rather difficult, since other catalysts [18] or materials [14] (fibers) were utilized. In the study of PET melts, Chen et al. [15] determined that the zinc acetate dihydrate amount in the reaction mixture has a linear dependency from 0.05 to 0.2 wt.%, while a further increase in catalyst content has no effect on reaction rate, presumably because of low catalyst solubility. This is aligned with the fact that in their experiments, the EG-to-PET *w*/*w* ratio was 1.3, suggesting also a lower concentration of EG as a reactant and also lower availability of EG for catalyst dissolution. Thus, the difference in their study, in comparison to ours, could be a combined effect of lower solubility of the catalyst, since a lower EG-to-PET *w*/*w* ratio was used, and also a lower amount of produced BHET, since the EG-to-PET *w*/*w* ratio, as will be shown in the following text, influences the reactive equilibrium and final BHET yield.

#### 3.3.2. Ethylene Glycol-to-PET Ratio Effect

**Figure 10:**
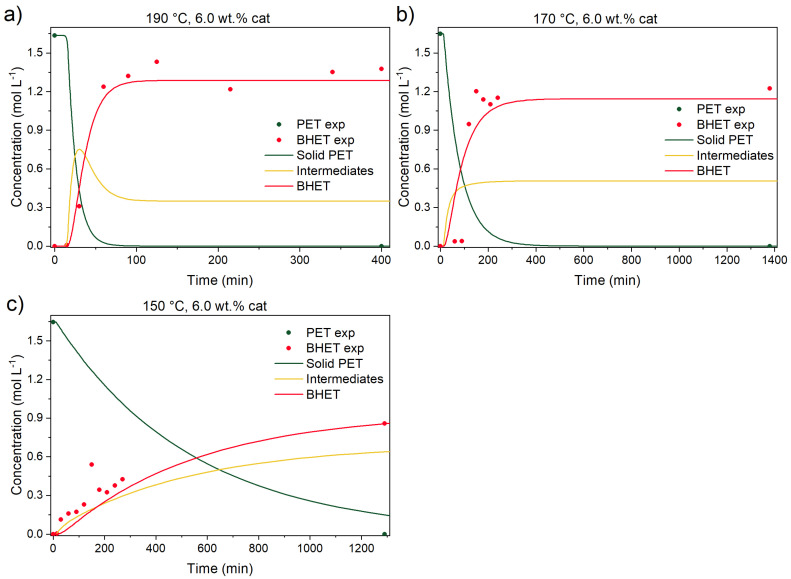
Catalyzed PET glycolysis at (**a**) 190 °C, (**b**) 170 °C, and (**c**) 150 °C. Reaction conditions: EG-to-PET *w*/*w* ratio of 2.5, 1000 rpm, and 6.0 wt.% of added catalyst. Reaction conditions are presented in Table S2, Entries 13, 16, and 18.

Another important aspect for a successful PET glycolysis, with the aim of achieving the highest PET conversion and subsequently BHET yield, is the ratio between EG and PET. These parameters also determine the productivity of the process and provide the important data for process design at scale-up. For this reason, the EG-to-PET *w*/*w* ratio of 2.5 has been studied, and the results are shown in Figure 10.

Here, the final BHET yield was 78%, which is 21% lower than the EG-to-PET *w*/*w* ratio of 5. The data suggests that intermediates and BHET are in a reactive equilibrium, as suggested by the kinetic model. A similar observation was made by a previous study [14], where the increase in the EG-to-PET *w*/*w* ratio from 5 to 7 improves the BHET yield from ~84 to 92%, respectively. In our case, the optimal ratio was lower, which could be the consequence of different sample preparation and analysis after the reaction. Additionally, PET conversion in their case was lower, while in our case, there was no solid residue in the reactor after the reaction at 190 °C. In another study [18], an EG-to-PET *w*/*w* ratio of 2.4 was used, and the highest BHET yield was 78%, which aligns perfectly with our results and confirms that equilibrium between oligomers, dimers, and BHET is formed.

#### 3.3.3. Experiments with Added Water

**Figure 11:**
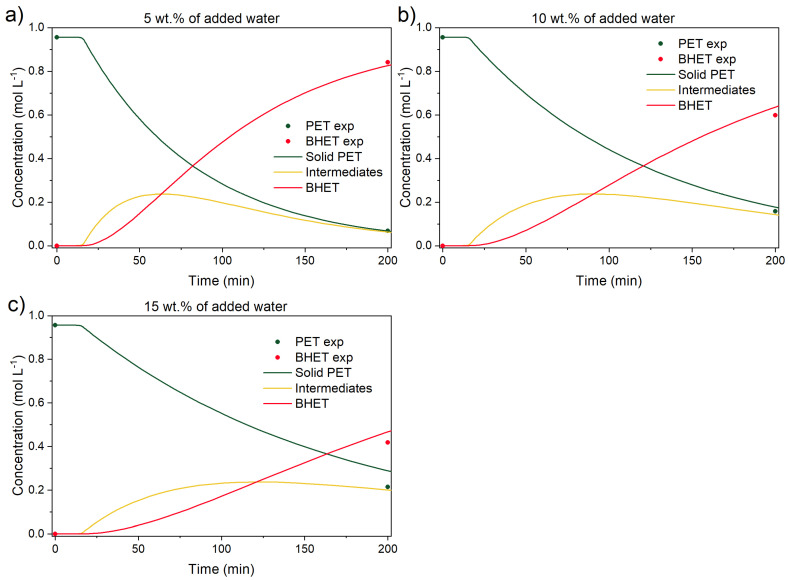
Catalyzed PET glycolysis with (**a**) 5, (**b**) 10, and (**c**) 15 wt.% of added water. Reaction conditions: 190 °C, EG-to-PET *w*/*w* ratio of 5, 1000 rpm, and 0.6 wt.% of added catalyst. Reaction conditions are presented in Table S2, Entries 44–46.

Another important parameter for a successful PET glycolysis, from an industrial standpoint, is the initial water content inside the reactor. PET flakes are usually washed prior to the reaction to remove the impurities. This means the flakes are usually also dried, causing another processing step in an industrial setting. For this reason, experiments with added water were tested to determine how much BHET is produced if the flakes are not completely dried before the reaction. In Figure 11 experiments with 5, 10, and 15 wt.% of added water are shown. During the reaction, the water vapor pressure increased, effectively preventing sampling during the reaction. Once the reactor was cooled, a sample was collected. The experimental procedure is described in detail in the ESI. Additionally, the kinetic model was updated to account for water content and the inclusion of an inhibition constant in all forward reactions. The modified equations are also provided in ESI.

From the figure above, water amount affects the final BHET yield. For example, BHET yield, at the same reaction conditions without added water (190 °C, EG-to-PET *w*/*w* ratio of 5, 1000 rpm, and 0.6 wt.% of added catalyst), was 99% of the theoretical BHET yield, calculated by Equation 2. In water-added experiments, the BHET yields were 88, 62, and 43% for 5, 10, and 15 wt.% of added water, respectively. In other words, 5 wt.% of added water lowers BHET yield by roughly 10%, while the addition of 15 wt.% of water lowers BHET yield by more than 50%. This suggests that water must be removed from the starting material to improve the overall conversion and BHET yield or, as is shown below, removed during the reaction through a reflux condenser.

#### 3.3.4. Kinetic Parameters, Validation Experiments, and Model Predictions

**Figure 12:**
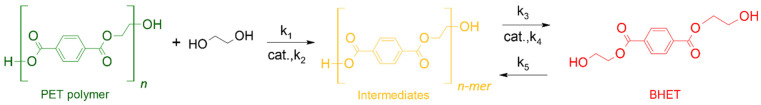
Proposed reaction mechanism for PET glycolysis. The color of each compound matches the colors in the concentration vs. time plots above.

From all experimental points above, where variation of temperature, catalyst amount, EG-to-PET *w*/*w* ratio, and water addition was performed, a kinetic model was assembled, and Table 1, Table 2 and Table 3 show the calculated kinetic parameters. In Figure 12 the proposed reaction mechanism for PET glycolysis is shown.

When comparing the forward *k*_1_ and *k*_2_ reaction rate constants, which are responsible for converting solid PET to intermediates, the results show that the catalyzed reaction rate is, as expected, much faster. Additionally, the order of the catalyst concentration *α* is 0.51, meaning that the concentration of the catalyst does not affect to a great extent the overall reaction rate; however, the catalyst has to be present inside the reaction mixture to improve the conversion, since *k*_2_ is faster than *k*_1_. Comparing *Ea*_2_ with the literature data, the determined activation energies are less consistent than expected. Lopez et al. [18] determined the *Ea* to be 185 kJ mol^−1^, which is higher than our *Ea*_2_; however, their model does not include the uncatalyzed reaction contribution, and also a different catalyst was used. In another study [15], where both reaction contributions (catalyzed and uncatalyzed) to the conversion of PET were included, the catalyzed and uncatalyzed activation energies were determined at 85 and 108 kJ mol^−1^, respectively.

Contrary to irreversible *r*_1_ and *r*_2_, reactions *r*_3_, *r*_4_, and *r*_5_ represent the reversible reaction network, where intermediates are converted to BHET. The forward reaction rate constants (*k*_3_ and *k*_4_) are higher than the backward reaction rate constant (*k*_5_). Again, the catalyzed reaction rates are much faster than the uncatalyzed ones, corresponding to experimental observations.

**Figure 13:**
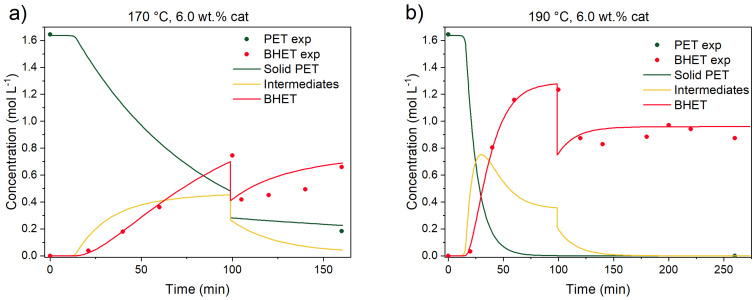
Validation experiment with the addition of fresh EG after 100 min of reaction at (**a**) 170 °C and (**b**) 190 °C. Reaction conditions: 1000 rpm, initial EG-to-PET *w*/*w* ratio of 2.5; after 100 min, the ratio is 5, with 6.0 wt.% of added catalyst. Reaction conditions are presented in Table S2, Entries 47 and 48.

To validate the kinetic model, two validation experiments were performed. First, to test the reversibility of the model, an experiment with added EG was performed at 190 °C. The experiment was initiated with EG-to-PET *w*/*w* ratio of 2.5, and after the concentration of BHET reached equilibrium, fresh EG was added to the reaction mixture to shift the EG-to-PET *w*/*w* ratio to 5, and the reaction was performed for an additional 150 min. Additionally, an experiment at 170 °C was performed, and, again, after 100 min of reaction, fresh EG was added. This is shown in Figure 13.

The model was modified to account for the reaction mixture’s volume change. In a classical experiment, the ODE15s solver is used to compute the concentration profiles from the initial conditions to a defined end time. In the modified version, the experiment begins at an EG-to-PET *w*/*w* ratio of 2.5. Integration is carried out up to the point just before fresh EG is introduced into the reactor. At this point, the integration is paused, and a new simulation is initiated using updated boundary conditions. Practically, the concentration of each compound is adjusted by multiplying it by the previous volume and dividing it by the new total volume (initial volume + added EG volume). This can be seen as a drop in the concentration profile for all compounds after the addition of EG. As can be seen from the figure above, when the reaction was performed at 190 °C, after the addition of EG, the predicted concentration for BHET drops but then gradually rises to reach the same BHET yield as in an experiment where the EG-to-PET *w*/*w* ratio of 5 was set from the beginning.

**Figure 14:**
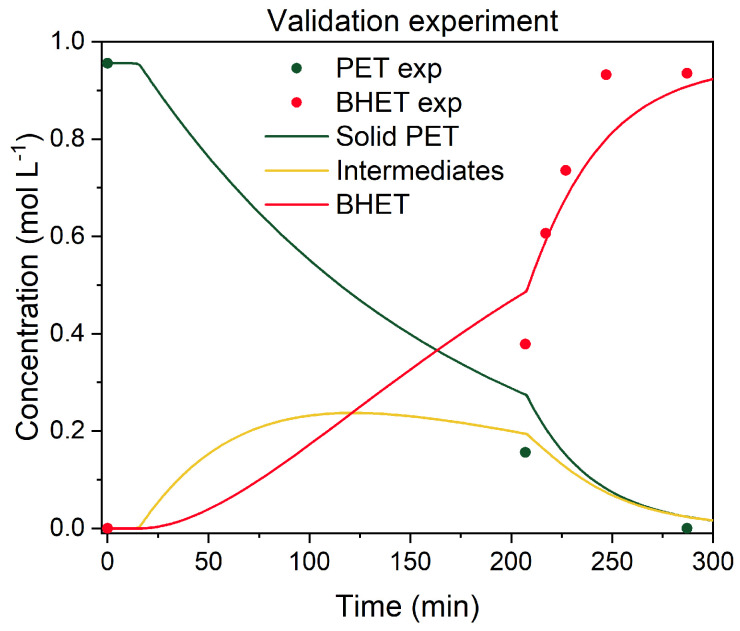
Validation experiment with dynamic water removal after 200 min of the reaction. Reaction conditions: 190 °C, 1000 rpm, EG-to-PET *w*/*w* ratio of 5, 1000 rpm, 0.6 wt.% of added catalyst, and 15 wt.% of added water. Reaction conditions are presented in Table S2, Entry 49.

The second validation experiment was performed to test if water could be removed from the reaction mixture during the reaction, excluding the need to dry the PET material prior to glycolysis. For this reason, the experiment was performed in the following manner: The reaction was initiated at 190 °C, with an EG-to-PET *w*/*w* ratio of 5, 0.6 wt.% of catalyst, 1000 rpm, and 15 wt.% of added water. After 200 min of reaction, the gas phase was released through the gas exhaust port to allow the removal of water vapor out of the reactor. Since there was no water left in the mixture, the pressure rise was no longer a problem, allowing normal sampling. This can be seen in Figure 14.

After 200 min, when water was removed from the reactor and there was no water inhibition left, the predicted concentration profiles for solid PET and BHET described the experimentally determined with good accuracy. This is a very important observation from an industrial point of view, because it shows that rather than PET drying prior to glycolysis, water distillation is required to remove the water vapors during the reaction to achieve the same BHET yield as with dried PET material.

**Figure 15:**
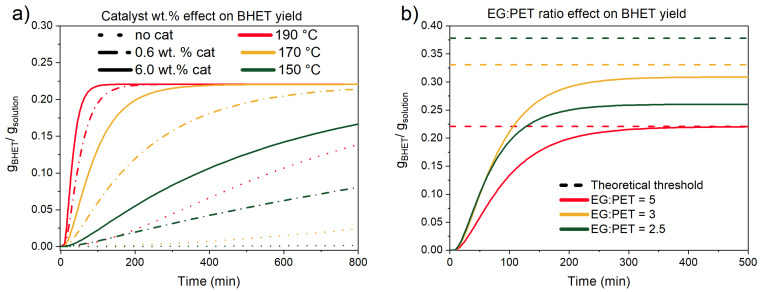
Kinetic model BHET yields prediction profiles. (**a**) catalyst variation at three temperatures and EG-to-PET *w*/*w* ratio of 5. (**b**) EG-to-PET *w*/*w* ratio variation and comparison with theoretical yield, calculated by Equation 2 at 170 °C, 6.0 wt.%.

Lastly, the kinetic model was utilized to generate g_BHET_/g_solution_ profiles for general comparison on how two of the most important parameters, namely catalyst amount and EG-to-PET *w*/*w* ratio, influence BHET yield (Figure 15).

It can be seen that catalyst addition greatly improves the reaction rate and, consequently, shortens reaction time. However, if removing the catalyst is problematic or undesired, the same BHET yield can be obtained without catalyst addition at the cost of a longer reaction time. Next, EG-to-PET *w*/*w* ratio is very detrimental to BHET yield. Our investigation shows that higher mass ratios than 3 are required to achieve BHET yields higher than 99%. Additionally, if a continuous system was developed, the exiting solution with dissolved BHET and oligomers can be mixed with additional EG to boost the conversion towards BHET and improve the final purity of the product.

## 4. Conclusions and Outlook

PET glycolysis is an important plastic recycling reaction, where PET material is converted to initial building blocks, which could be used in the PET synthesis process again. Our investigation revealed important mechanistic insights into the glycolysis reaction, elucidating how crucial parameters such as temperature, EG-to-PET *w*/*w* ratio, stirring speed, flake particle size, and catalyst concentration affect the final BHET yield. The main conclusions are the following:

Solid PET material does not dissolve and depolymerize, but depolymerization to oligomers and dimers occurs directly from solid particles.

While the presence of the catalyst increases the conversion rate immensely, its loading has no impact on the conversion of intermediates.

The temperature of the reaction has a strong impact on the conversion rate of PET.

Experiments suggest that there is no external mass transfer resistance, meaning that stirring rate does not affect the final yield of BHET.

Smaller flake size does not improve conversion rate.

Dynamic experiments with the addition of EG improve the final BHET yield.

Water inhibits conversion of PET reversibly, meaning that if water is distilled from the reaction mixture, the final BHET yield is the same as if dry flakes were used.

Most of these conclusions are also beneficial for practical application in industrial PET recycling. For example, the lack of particle size influence on BHET conversion means less preprocessing of PET flakes, the low influence of stirring means lower energy consumption, and the understanding of water consistency offers flexibility in processing PET raw materials with reduced drying processes.

Since stirring rate at the laboratory scale did not affect the conversion rate of PET, this phenomenon should also be tested at larger-scale reactors to determine how long the glycolysis in an unstirred reactor would take. Moreover, future research should focus on scaling up the glycolysis process by designing a continuous reactor system with in situ removal of BHET to drive reaction equilibrium and improve yields. In addition, since stirring did not affect the glycolysis rate, future studies should also address novel reactor designs for industrial applications. For example, a continuous trickle bed reactor with continuous addition of solid PET flakes and EG (fresh + the one collected at the outlet) would in theory allow a continuous process, where the liquid phase would be constantly removed from the reactor, separating EG and BHET and returning EG to the reactor.

Catalyst optimization, including the development of greener and reusable alternatives, should be prioritized to enhance efficiency and sustainability. Further studies are needed to explore the impact of mixed or contaminated PET feedstocks, as well as the effects of dyes, fillers, and coatings on reaction performance. Economic and environmental assessments, including techno-economic analysis (TEA) and life cycle assessment (LCA), are also crucial to evaluate the feasibility and sustainability of industrial-scale implementation.

Ultimately, our work demonstrates a significant step forward in understanding and optimizing PET glycolysis, reaffirming our ability to tackle the challenges of plastic recycling with innovative solutions.

## Figures and Tables

**Table 1:** Kinetic parameters for uncatalyzed reactions. Reaction constants have been determined at 170 °C.

Uncatalyzed Reaction Rate Constants and Corresponding Activation Energies					
*k*_1_ [min^−1^]	*k*_3_ [min^−1^]	*k*_5_ [min^−1^]	*Ea*_1_ [kJ mol^−1^]	*Ea*_3_ [kJ mol^−1^]	*Ea*_5_ [kJ mol^−1^]
(5.17 ± 0.5) × 10^−4^	0.0010 ± 0.00034	(2.88 ± 0.4) × 10^−7^	180 ± 23	57 ± 12	<5

**Table 2:** Kinetic parameters for catalyzed reactions. Reaction constants have been determined at 170 °C.

Catalyzed Reaction Rate Constants and Corresponding Activation Energies			
*k*_2_ [L*^α^* mol^−*α*^ min^−1^]	*k*_4_ [L^β^ mol^−β^ min^−1^]	*Ea*_2_ [kJ mol^−1^]	*Ea*_4_ [kJ mol^−1^]
0.06 ± 0.005	0.039 ± 0.004	158 ± 6	37 ± 6

**Table 3:** Catalyst concentration order for catalyzed reactions and EG-to-PET *w*/*w* ratio parameters.

Catalyst Concentration Order		Ratio Parameter	Water Inhibition
*α*	β	S_1_	Inhib
0.51 ± 0.0	5.43 ± 0.16 × 10^−6^	27.6 ± 0.1	0.34 ± 0.04

## Data Availability

The data presented in this study are available from the corresponding author upon request.

## Supplementary Materials

The following supporting information can be downloaded at: https://www.mdpi.com/article/10.3390/polym17162246/s1, Figure S1: Calibration curves for BHET (left) and MHET (right). This calibration curve is for ACN method. Figure S2: Reproducibility of the experiments of PET glycolysis at 3 different temperatures for 0.5 wt.% catalyst at an EG-to-PET ratio of 5 after 60 min of reaction. Each bar represents the average of three independent experiments, with error bars showing the standard deviation. Figure S3: Catalyzed PET glycolysis at 190 °C—(a) 0.6, (b) 3.0, (c) 6.0 wt.% catalyst, short reaction time experiments for SEC analysis. Reaction conditions: EG-to-PET *w*/*w* ratio of 5, 1000 rpm. Figure S4: Catalyzed PET glycolysis at 170 °C—(a) 0.6, (b) 3.0, (c) 6.0 wt.% catalyst, short reaction time experiments for SEC analysis. Reaction conditions: EG-to-PET *w*/*w* ratio of 5, 1000 rpm. Figure S5: Catalyzed PET glycolysis at 150 °C—(a) 0.6, (b) 3.0, (c) 6.0 wt.% catalyst, short reaction time experiments for SEC analysis. Reaction conditions: EG-to-PET *w*/*w* ratio of 5, 1000 rpm. Table S1: Average molecular weight of PET standard calculated on calibration curve of PMMA. Table S2: Table with all preformed experiments on Amar reactor systems. Table S3: SEC experiments and results of SEC experiments, solid residue after the end of reaction, mass of BHET analysed on HPLC.

## Abbreviations

**Abbreviation**	**Definition**
BHET	Bis(2-hydroxyethyl) terephthalate
DMT	Dimethyl terephthalate
EG	Ethylene glycol
HFIP	1,1,1,3,3,3-Hexafluoro-2-propanol
HPLC	High-performance liquid chromatography
MHET	Monohydroxyethyl terephthalate
NaTFA	Sodium trifluoroacetate
PET	Polyethylene terephthalate
PDI	Polydispersity index
PMMA	Polymethyl methacrylate
RI	Refractive index
SEC	Size exclusion chromatography
TPA	Terephthalic acid
