# Supplementary material for: Polyethylene Terephthalate Glycolysis: Kinetic Modeling and Validation
Source: Polymers (Basel). 2025 Aug 20;17(16):2246. doi: 10.3390/polym17162246 (PMC12389461; doi:10.3390/polym17162246)
Supplement: Supplementary file 1 [file polymers-17-02246-s001.zip › polymers-3789670-supplementary.pdf]

Supporting Information for the Research Article:

Polyethylene Terephthalate Glycolysis: Kinetic Modeling and Validation

Maja Gabrič†[1,2], Žan Lavrič†[1,2*], Martin Schwiderski[3], Laureline Marc[3], Erik Temmel[3], Miha Grilc[1,2] and Blaž Likozar[1,4]

[1] Department of Catalysis and Chemical Reaction Engineering, National Institute of Chemistry, Hajdrihova 19, SI-1000 Ljubljana, Slovenia

[2] University of Nova Gorica, Vipavska 13, SI-5000 Nova Gorica, Slovenia

[3] Sulzer Chemtech Ltd., Neuwiesenstrasse 15, 8401 Winterthur, Switzerland

[4] Faculty of Polymer Technology, Ozare 19, SI-2380 Slovenj Gradec

† Authors contributted equally to the manuscript

Contents

1. Development of analytical methods 2

1.1 HPLC method 2

1.1.1 Sample preparation for HPLC 3

1.2. Size exclusion chromatography 4

1.2.1. Calibration curve for PET samples 4

1.2.1. Sample preparation for SEC 4

2. PET glycolysis experiments 4

2.1 Experiments for SEC analysis of PET glycolysis products 4

2.2 Experiments on the influence of initial PET material size 5

2.3 Experiments tailored for influence of external mass transfer limitations 5

2.4 Experiments with added water 5

3. Modified kinetic model for water removal 6

Supplementary tables and figures 8

1. Materials and chemicals

The following chemicals and materials were used in this study, along with their respective suppliers: Poly(ethylene terephthalate) (PET) flakes were provided by Sulzer Chemtech Ltd.; ethylene glycol (EG, 99%) was purchased from Sigma Aldrich, Germany; zinc acetate dihydrate was obtained from Merck, Germany; and nitrogen gas (N_2) for reactor purging was supplied by Messer, Germany. For Size Exclusion Chromatography (SEC), 1,1,1,3,3,3-Hexafluoro-2-propanol (HFIP) and sodium trifluoroacetate (NaTFA) were both sourced from Sigma Aldrich, Germany. SEC calibration employed PET molecular weight standards and polymethyl methacrylate (PMMA) standards, both purchased from PSS Polymer Standard Service, Agilent, Germany.

2. Development of analytical methods

2.1 HPLC method

The analysis and quantification of bis(2-hydroxyethyl) terephthalate (BHET) and 4-((2-Hydroxyethoxy) carbonyl) benzoic acid (MHET) was performed with High Performance Liquid Chromatography (HPLC). The instrument used was Ultimate 3000, ThermoFisher Scientific with C18 Ascentis express

90 Å C18 column (15 cm x 4.6 mm, 5 µm) and with 5 µm Guard Column (Supelco, Merck). The best separation of compounds of interest was achieved with the method that involved 1.0 mL min^{-1} flow rate of 30:70 ratio of ultrapure Milli Q water and Methanol (Methanol gradient grade for HPLC, Sigma Aldrich, USA). The column was kept at 30 °C and the injection volume was 1 µL. The detector of choice was UV-VIS and all the selected compounds were monitored with wavelength of 254 nm. BHET from Sigma Aldrich with purity 96% and MHET from BLD pharm with 95% purity was used for calibration (**Figure S1**). Standards were dissolved with different concentrations in Mobile phase. The calibration curves were designed to encompass a range, from 20 mg L^{-1} to 200 mg L^{-1} for BHET and 5 mg L^{-1} to 50 mg L^{-1} for MHET. To quantify additional peaks, oligomers and dimers, besides BHET and MHET, we employed HPLC coupled with Mass Spectrometry (LC-MS). LC–MS analysis was performed on an UltiMate 3000 UHPLC system (Thermo Scientific, U.S.A.) coupled with a triple quadrupole/linear ion trap mass spectrometer (4000 QTRAP LC–MS/MS System; Applied Biosystems/MDS Sciex, Ontario, Canada). For the LC-MS method, a mobile phase flow rate of 1 mL min^{-1} was employed, consisting of a 30:70 ratio of ultrapure Milli Q water to Methanol, with the addition of 0.1 % formic acid (Formic acid, Sigma Aldrich, USA).

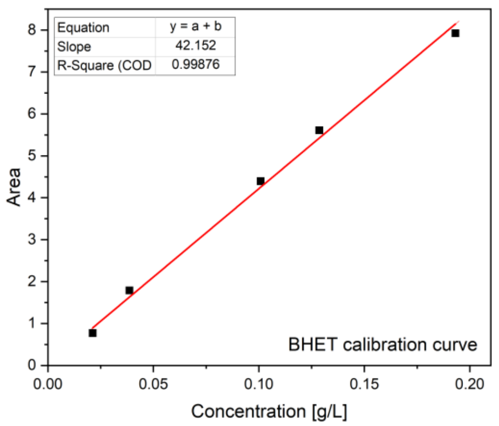

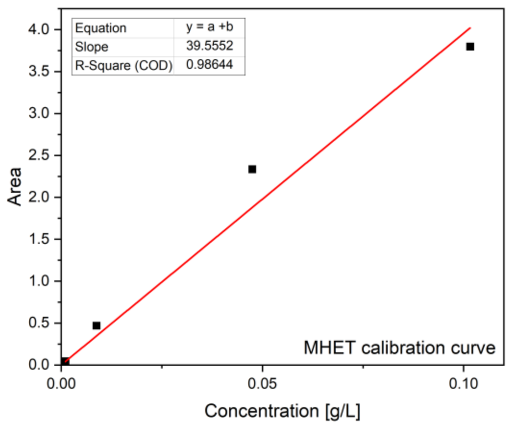

Figure S1 Calibration curves for BHET (left) and MHET (right). This calibration curve is for ACN method.

2.1.1 Sample preparation for HPLC

The sample for HPLC method was volumetrically diluted promptly after withdrawal from the reactor, while still in hot and liquid state. Samples were diluted with the mobile phase for HPLC method. Usual dilution was performed in two-steps: first step involved initial 100 x dilution of the original sample (990 µL of solvent and 10 µL of sample), followed by a second step of 10 x-20 x dilution, depending on the expected BHET concentration. To ensure the consistency and reproducibility of the sample preparation, dilutions were performed in parallel. Subsequently, the samples underwent filtration using 0.2 µm PTFE filters. Immediate analysis of the samples was conducted within a 3 hour timeframe. On occasions when HPLC was temporarily unavailable, diluted samples were stored in the refrigerator.

Upon reanalysis after one month of refrigeration, the results demonstrated repeatability, confirming the stability of the samples over the storage period.

2.2 Size exclusion chromatography

2.2.1 Calibration curve for PET samples

In polymers, the molecular weight is not a discrete value, but a distribution. To find out how many molecules there are at each molecular weight, we need to calculate the average number. This is the sum of the molecular weights of all polymer chains present in the polymer divided by the number of all polymer chains present in the polymer. Due to the insolubility of high molecular weight PET standards, which were delivered in large granules and could not be dissolved in mobile phase, an alternative calibration curve was created from PMMA standards. Further, PMMA standards also possess similar chemical structure to the PET, so margin of error is minimized. To prove that the PMMA calibration curve is suitable for the quantification of PET samples, PET standards with lower molecular weights were measured and used with the PMMA calibration curve. The results, shown in **Table S1** indicate that the margin of error in the determination of PET molecular weights with the PMMA standards is approximately 25 % underestimated. This demonstrates the acceptable accuracy of SEC technique and justifies the use of PMMA calibration over the compromised dissolution of high M_w PET standards.

Table S1 Average molecular weight of PET standard calculated on calibration curve of PMMA.

	**Actual M_w of PET from declaration [Da]**	**M_w calculated from calibration with PMMA [Da]**
PET STD 1	3470	2648
PET STD 2	9870	6763

1.2.1. Sample preparation for SEC

Solid residues of PET were dissolved in the mobile phase for SEC, composed of HFIP and 20 mM NaTFA, at a concentration of 20 mg mL^{-1}. The dissolution process involved magnetic stirring to ensure homogeneity in the solution. Subsequently, the samples underwent filtration through 0.45 μm PTFE filters before being subjected to analysis.

3. PET glycolysis experiments

3.1 Experiments for SEC analysis of PET glycolysis products

The experiments to obtain representative samples for SEC were carried out with Amar reactors in the same way as described earlier, while reaction was stopped after 1 hour, and solid PET residue was collected at various reaction conditions. All three variations of temperature and catalyst wt.% were tested

at ratios of 2.5 and 5.0. After the completion of the experiment, the reaction mixture underwent hot filtration through a coarse steel mesh at 60 °C, ensuring effective separation of residual plastic particles. Subsequently, the obtained solid residue was washed with methanol to dissolve any remaining BHET. Following the washing step, the solid residue was dried overnight in the oven at 60 °C. The resulting dried solid residues were weighted and used for SEC analysis.

3.2 Experiments on the influence of initial PET material size

To observe how initial size of PET material influences the BHET production, the PET flakes were shredded with the Reutsch cutting mill. The flakes were shredded into 3 different sizes. 1 mm – 2 mm, 2 mm-3.15 mm and > 3.15 mm. The collected fractions were later used in experiments at 170 °C at a fixed EG-to-PET w/w ratio of 5 and a catalyst content of 0.1 wt.% (100 g of EG, 20 g of PET, 0.12 g of zinc acetate dihydrate).

3.3 Experiments tailored for influence of external mass transfer limitations

To further investigate the external mass transfer limitations, we carried out 3 experiments with 3 stirring speeds, namely 100, 500 and 1000 rpm. The experiments were carried out under the following reaction conditions: 190 °C, EG/PET ratio of 5, 0.1 wt.% catalyst (100 g of EG, 20 g of PET, 0.12 g of zinc acetate dihydrate).

3.4 Experiments with added water

To evaluate the final yield of BHET, three trials were conducted with the addition of water. The trials were conducted with an addition of 5, 10 and 20 wt.% water per total weight of the reaction mixture. The tests with water were carried out in a sealed reactor to ensure water retention in the system. As the temperatures were above 100 °C, sampling was not possible as the water would evaporate from the system. For this reason, the reaction was started with an EG-to-PET w/w ratio of 5, 190 °C, 0.1 wt.% catalyst (100 g of EG, 20 g of PET, 0.12 g of zinc acetate dihydrate 1000 rpm and 5, 10 or 20 wt.% added water, corresponding to the addition of 6.4, 13.4 and 21 g of water to the original 120 g of EG and PET flakes. After 200 min, the reaction was stopped by rapid cooling to room temperature and a liquid sample was discarded to determine the final BHET yield.

4. Reproducibility of experiments

To show that our data is reproducible, we have performed 3 different experiments for different reaction temperatures (190 °C, 170 °C, and 150 °C) using 0.5 wt.% catalyst at an EG-to-PET w/w ratio of 5 after 60 min of reaction at 1000 rpm stirring speed. The experimental procedure was the same as for glycolysis experiments (Manuscript Section 2.1). For quantification of BHET and MHET after the glycolysis the High-Performance Liquid Chromatography.

For each temperature, three independent experiments were carried out, and the bars represent the average $g_{BHET}/g_{solution}$ values, with error bars indicating the standard deviation. The yields were 0.2005±0.0097, 0.0186±0.0010, 0.0019±0.0002 for 190 °C, 170 °C, and 150 °C, respectively. The small standard deviations confirm good reproducibility of the measurements under the same reaction conditions.

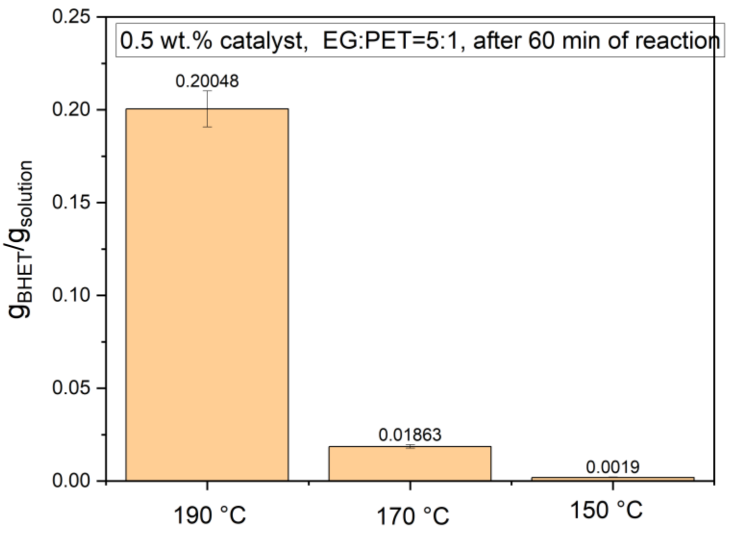

Figure S2: Reproducibility of the experiments of PET glycolysis at 3 different temperatures for 0.5 wt.% catalyst at an EG-to-PET ratio of 5 after 60 min of reaction. Each bar represents the average of three independent experiments, with error bars showing the standard deviation.

5. Modified kinetic model for water removal

The kinetic model described herein includes minor additions in some equation terms, to include water addition. For this reason, all equations from the manuscript are written here with the added terms, which marked with red color.

Equations **1, 2, 3** and **4** were improved with the term for inhibition with water. Inhib is a new derived constant, which was incorporated in the GUI extension.

Equation 1

$$r_1 = \frac{k_1 \cdot C_{\text{solid PET}}}{1 + inhib \cdot C_{\text{water}}}$$

Equation 2

$$r_2 = \frac{k_2 \cdot C_{\text{solid PET}} \cdot C_{\text{catalyst}}{}^{\alpha}}{1 + inhib \cdot C_{\text{water}}}$$

Equation 3

$$r_3 = \frac{k_3 \cdot C_{\text{Intermediates}}}{1 + inhib \cdot C_{\text{water}}}$$

Equation 4

$$r_4 = \frac{k_4 \cdot C_{\mathrm{Intermediates}} \cdot C_{\mathrm{catalyst}}{}^{\beta}}{1 + inhib \cdot C_{\mathrm{water}}}$$

Equation 5

$$r_5 = k_5 \cdot C_{\mathrm{BHET}} \cdot exp\left(S_1 \cdot \left(\frac{1}{ratio} - \frac{1}{5}\right)\right)$$

Mass balance equations for reversible model are shown with **Eq. Equation 6** –**Equation 8**:

Equation 6

$$\frac{\mathrm{d}C_{\mathrm{solid\ PET}}}{\mathrm{d}t} = -r_1 - r_2$$

Equation 7

$$\frac{\mathrm{d}C_{\mathrm{Intermediates}}}{\mathrm{d}t} = +r_1 + r_2 - r_3 - r_4 + r_5$$

Equation 8

$$\frac{\mathrm{d}C_{\mathrm{BHET}}}{\mathrm{d}t} = +r_3 + r_4 - r_5$$

Equation 9

$$\frac{\mathrm{d}C_{\mathrm{water}}}{\mathrm{d}t} = -k_L \cdot A \cdot (C_{\mathrm{H_2O,liq}} - C_{\mathrm{H_2O,vap}})$$

Equation 10

$$C_{H_2O,vap} = \frac{P_{\mathrm{H_2O}}}{R \cdot T}$$

Equation 9 and **Equation 10** were added as a balance for water inside the liquid reaction mixture. Because the reaction temperature of the dynamic experiment, shown in **Figure 13**, is much higher than the boiling point of water, the parameters k_{L} and A were assumed to be extremely high, when the effluent of the gas phase was opened, to allow water removal from the reactor. In practice, when we opened the outlet port and water vapor pressure was released, the effective removal of all water from the liquid phase occurred in < 2 min, which allowed us to set the constants at high values to and simplify the kinetic modelling. In future experiments, this will be addressed and scaled to certain reactor design.

Supplementary tables and figures

Table S2 Table with all preformed experiments on Amar reactor systems.

Entry	Aim of the experiment	Material used	rpm	EG-to-PET w/w ratio	t (reac) [min]	T (reac) [°C]	Catalyst wt.% per PET mass
1	Various temperatures and wt.% of catalyst for BHET analysis.	PET mixed flakes	1000	5	1440	190	6.0
2		PET mixed flakes	1000	5	855	190	3.0
3		PET mixed flakes	1000	5	360	190	0.6
4		PET mixed flakes	1000	5	5110	190	/
5		PET mixed flakes	1000	5	1200	170	6.0
6		PET mixed flakes	1000	5	1210	170	3.0
7		PET mixed flakes	1000	5	1345	170	0.6
8		PET mixed flakes	1000	5	4733	170	/
9		PET mixed flakes	1000	5	1205	150	6.0
10		PET mixed flakes	1000	5	2645	150	3.0
11		PET mixed flakes	1000	5	2980	150	0.6
12		PET mixed flakes	1000	5	4733	150	/
13	Various temperatures and wt.% of catalyst for BHET analysis.	PET mixed flakes	1000	2.5	460	190	6
14		PET mixed flakes	1000	2.5	360	190	3.0
15		PET mixed flakes	1000	2.5	180	190	0.6
16		PET mixed flakes	1000	2.5	1500	170	6.0
17		PET mixed flakes	1000	2.5	270	170	3.0
18		PET mixed flakes	1000	2.5	1290	150	6.0
19*	Various temperatures and wt.% of catalyst for SEC - RATIO 1:5	PET mixed flakes	1000	5	60	190	6.0
20*		PET mixed flakes	1000	5	60	190	3.0
21*		PET mixed flakes	1000	5	60	190	0.6
22*		PET mixed flakes	1000	5	60	170	6.0
23*		PET mixed flakes	1000	5	60	170	3.0
24*		PET mixed flakes	1000	5	60	170	0.6
25*		PET mixed flakes	1000	5	60	150	6.0
26*		PET mixed flakes	1000	5	60	150	3.0
27*		PET mixed flakes	1000	5	60	150	0.6
28*	Various temperatures and wt.% of catalyst for SEC - RATIO 1:2,5	PET mixed flakes	1000	2.5	60	190	6.0
29*		PET mixed flakes	1000	2.5	60	190	3.0

30*		PET mixed flakes	1000	2.5	60	190	0.6
31*		PET mixed flakes	1000	2.5	60	170	6.0
32*		PET mixed flakes	1000	2.5	60	170	3.0
33*		PET mixed flakes	1000	2.5	60	170	0.6
34*		PET mixed flakes	1000	2.5	60	150	6.0
35*		PET mixed flakes	1000	2.5	60	150	3.0
36*		PET mixed flakes	1000	2.5	60	150	0.6
37		shredded flakes (1 mm - 2 mm)	1000	5	200	170	0.6
38	Various particle size	shredded flakes (>3.15 mm)	1000	5	200	170	0.6
39		shredded flakes (2 mm - 3.15 mm)	1000	5	200	170	0.6
40		PET mixed flakes	0	5	200	190	0.6
41	Various stirring speed	PET mixed flakes	100	5	200	190	0.6
42		PET mixed flakes	500	5	200	190	0.6
43		PET mixed flakes	1000	5	200	190	0.6
44		PET mixed flakes + **5 wt.% of dH_2O**	1000	5	200	190	0.6
45	Addition of water	PET mixed flakes + **10 wt.% of dH_2O**	1000	5	200	190	0.6
46		PET mixed flakes + **15 wt.% of dH_2O**	1000	5	200	190	0.6
47	Validation expetiments with addition of fresh EG	PET mixed flakes + **addition of fresh EG**	1000	5	200	170	6.0
48			1000	5	300	190	6.0
49	Validation experiment with water removal	PET mixed flakes + **15 wt.% water removal**	1000	5	300	190	0.6

* The experiments for the SEC experiments. The results and experiment conditions of SEC experiments are also represented in the Table S3

Table S3 SEC experiments and results of SEC experiments, solid residue after the end of reaction, mass of BHET analysed on HPLC.

Experiment conditions						Filtered PET solid residue after the end of reaction	Liquid product after reaction	Filtered PET solid residue after the end of reaction analyzed with SEC method		
**#**	**Reference to the entry in the Table S2**	**EG-to-PET w/w ratio**	***T* [°C]**	**wt. % of catalyst**	***t* [min]**	**m_{PET} [g]**	**m_{BHET} [g]**	**M_w [Da]**	**M_n[Da]**	***PDI***
Initial sample	/	/	/	/	/	/	/	63635	20214	3.15
A	19	5	190	6.0	60	2.7	20.0	8791	3724	2.36
B	20	5	190	3.0	60	3.3	21.9	9524	3407	2.80
C	21	5	190	0.6	60	6.0	25.2	10903	4157	2.62
D	22	5	170	0.06	60	12.4	9.5	30035	4781.6	6.28
E	23	5	170	3.0	60	16.8	5.4	22583	6453	3.50
F	24	5	170	0.6	60	18.0	2.1	24144	7034	3.43
G	25	5	150	6.0	60	19.5	0.7	35015	10099	3.47
H	26	5	150	3.0	60	20.0	0.4	36608	11259	3.25
I	27	5	150	0.6	60	19.7	0.2	33525	11054	3.03
J	28	2.5	190	6.0	60	1.4	32.9	8832	3717	2.38
K	29	2.5	190	3.0	60	4.2	33.6	9123	4182	2.18
L	30	2.5	190	0.6	60	12.5	16.7	14330	4848	2.96
M	31	2.5	170	6.0	60	24.3	7.0	17950	5705	3.15
N	32	2.5	170	3.0	60	25.3	4.8	21969	6677	3.29
O	33	2.5	170	0.6	60	26.7	5.4	21558	7127	3.20
P	34	2.5	150	6.0	60	28.6	1.7	31707	10089	3.14
R	35	2.5	150	3.0	60	29.0	1.3	29539	9611	3.11
S	36	2.5	150	0.6	60	29.6	0.6	34188	11092	3.08

Figures S3– S5 show the concentration *vs.* time profiles, which were also use within regression analysis for kinetic parameter estimation. These experiments are actually the short reaction time experiments and the residual solid PET was used for SEC analysis.

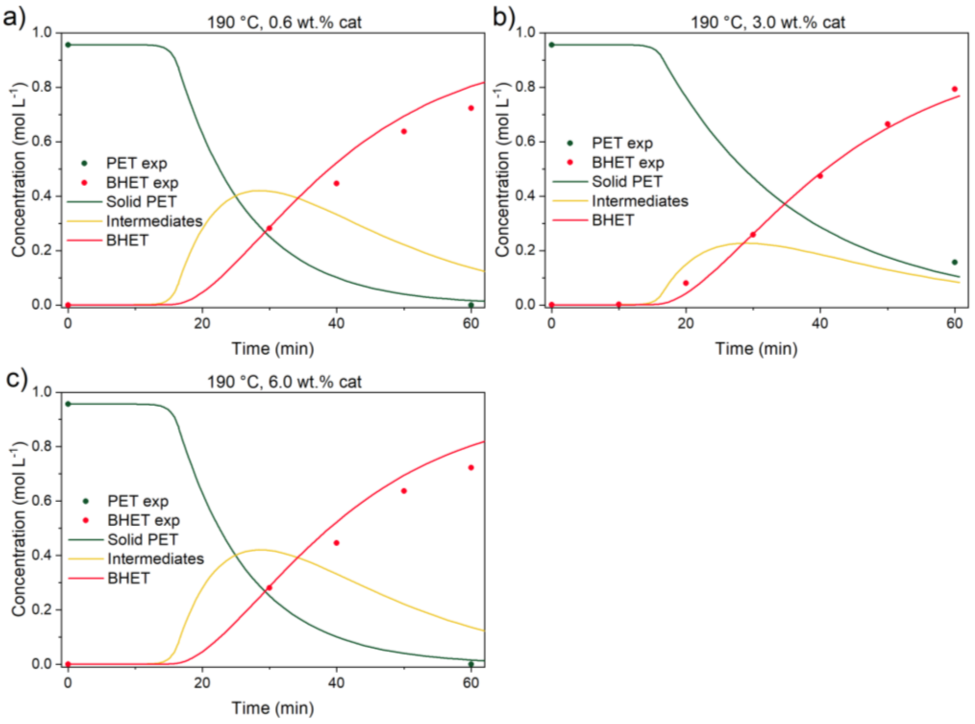

Figure S3 Catalyzed PET glycolysis at 190 °C – a) 0.6, b) 3.0, c) 6.0 wt. % catalyst, short reaction time experiments for SEC analysis. Reaction conditions: EG-to-PET w/w ratio of 5, 1000 rpm.

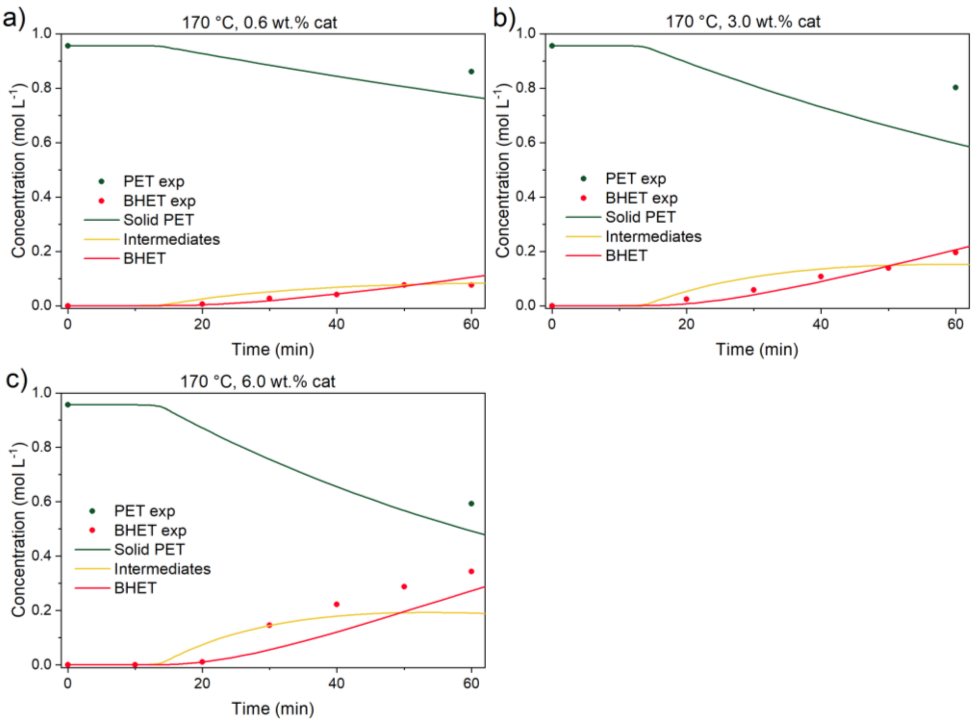

Figure S4 Catalyzed PET glycolysis at 170 °C – a) 0.6, b) 3.0, c) 6.0 wt. % catalyst, short reaction time experiments for SEC analysis. Reaction conditions: EG-to-PET w/w ratio of 5, 1000 rpm.

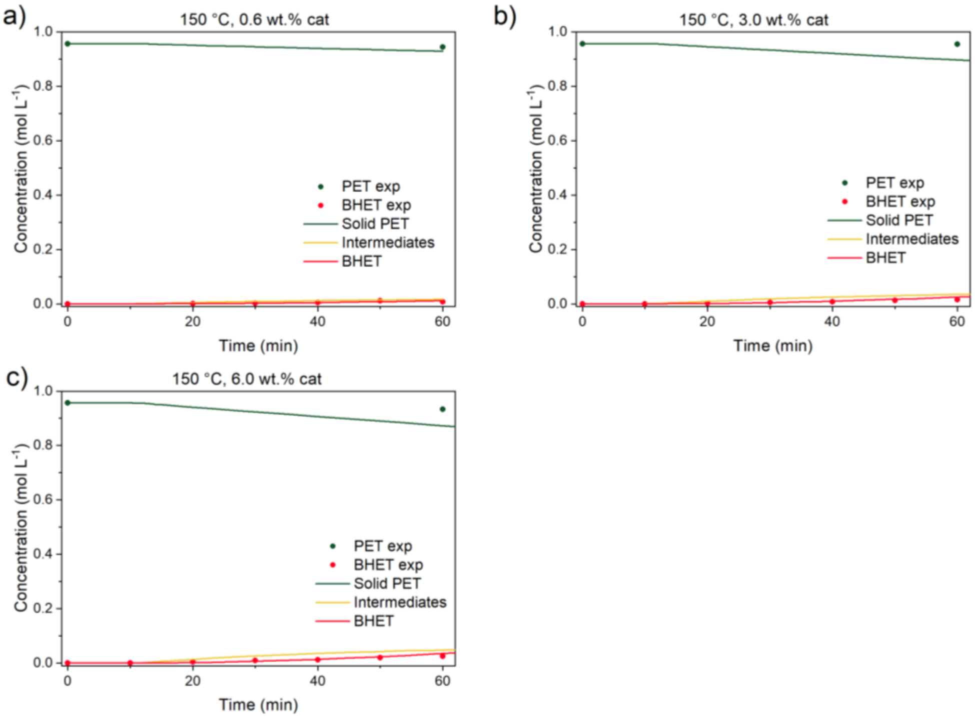

Figure S5 Catalyzed PET glycolysis at 150 °C – a) 0.6, b) 3.0, c) 6.0 wt. % catalyst, short reaction time experiments for SEC analysis. Reaction conditions: EG-to-PET w/w ratio of 5, 1000 rpm.
